# A 25-Year Bibliometric Analysis of Allergic Rhinitis Publications from Turkey

**DOI:** 10.4274/tao.2020.6076

**Published:** 2021-03-26

**Authors:** Cüneyt Orhan Kara, Erdem Mengi, Kübra Aktan, Bülent Topuz

**Affiliations:** 1 Department of Otorhinolaryngology, Pamukkale University Faculty of Medicine, Denizli, Turkey

**Keywords:** Allergic rhinitis, publication, bibliometrics, PubMed®, TRDizin®, otorhinolaryngology, Turkey

## Abstract

**Objective:**

To analyze the change over the 25 years period in the number of publications on allergic rhinitis from Turkey, and to compare the data of the four major relevant specialties.

**Methods:**

A search was conducted over 25-years between1994 and 2019 using the keywords “allergic rhinitis” and “Turkey” in PubMed^®^, and “*allerji*”, “*alerji*” and “*rinit*” in TRDizin^®^. The articles were grouped by specialty; namely, “Otorhinolaryngology (ORL),” “Pediatrics,” adult “Pulmonary disease” and adult “Allergy/immunology,” based on the affiliation of the first author. The total number of publications in each specialty group within the 25-year period were compared using a significance test for a difference in two proportions within the statistical assessment.

**Results:**

The 25-year results revealed 624 and 213 publications in the PubMed^®^ and the TRDizin^®^ databases, respectively. When the number of publications in a specific field in both databases was examined, the highest number of publications were identified in the ORL group, followed by the Pediatrics group. The number of publications in the ORL group was statistically higher than those in the “Pulmonary diseases” and “Allergy/immunology” groups in both the PubMed^®^ and the TRDizin^®^ databases (p=0.0001).

**Conclusion:**

The analysis of the number of 25-year allergic rhinitis publications from Turkey revealed that the academic interest of otolaryngologists in allergic rhinitis was unaffected, despite the challenges experienced in practice, with an increasing number of publications noted. When the number of 25-year publications was examined, ORL recorded the highest number of publications among all specialties.

## Introduction

Allergic rhinitis (AR) is a common disease, reported to affect 5%–40% of the general population, with evidence of a rising prevalence (1). In Turkey, the prevalence of physician-diagnosed AR is 20.1%, whereas that of self-reported AR is 23.8% in urban areas and 18.4% in rural areas (2, 3). The importance of AR can be attributed to its prevalence, its impact on quality of life and work/school performance, and its association with other comorbidities (4). Given their prevalence and the morbidity burden, AR and otolaryngic allergies are among the most common fields of interest among otorhinolaryngology (ORL) specialists (5, 6).

In Turkey, too, AR has always been an area of interest for otolaryngologists (7). The number of studies on AR started to increase especially in the 1980s in our country, in line with the global trend. In those years, ORL training clinics began to perform prick tests and to apply immunotherapies as a standard practice in patients with AR, although this increased interest of otolaryngologists in AR has witnessed a gradual decline in recent years (7).

Otolaryngologists and academicians in our country, like their peers around the world, do not only diagnose and treat diseases, but also do research in their fields of interest and publish in national and international journals. There is a lack of data on whether the declining interest of otolaryngologists in AR found reflection in the number of publications on AR from Turkey. Besides otolaryngologists, AR is also a field of interest for specialists of pulmonology, pediatrics, and adult allergy/immunology This bibliometric study analyzes the change in the number of AR publications from Turkey over a 25-year period and determines the impact of the declining interest of otolaryngologists in AR on the number of publications they make. The study further assesses the change in AR publications by other specialties with an interest in the condition and compares the numbers of their publications with the numbers of those by otolaryngologists.

## Methods

The study assessed the publications made over the 25-year period from 1994 to 2019 and included in international and national databases, namely, the PubMed^®^(USA) and the TRDizin^®^(Turkey). The search in PubMed^®^ was conducted using the keywords “allergic rhinitis” and “Turkey,” while the search in TRDizin^®^ was conducted using the keywords “allerji,” “alerji” and “rinit.” Titles and abstracts of all identified articles were reviewed individually. In cases which the abstract lacked sufficient details, assessment was made by accessing the full text of the article. In addition to AR, the study included also articles with titles such as “immunotherapy and complications” considering such terms as being part of “otolaryngic allergy,” and articles that did not mention “allergic rhinitis” in their title but did refer to AR in the text. The study excluded articles that appeared in the search but were not related to AR.

Identified articles were classified into publication groups as “clinical” for studies on disease prevalence, diagnosis, treatment, etc., and “experimental” for studies on animals, and as “case presentations,” “reviews” and “guidelines/consensus” according to the method used. And finally, analysis was made over the total number of publications to assess all specialty groups.

**Figure 1:**
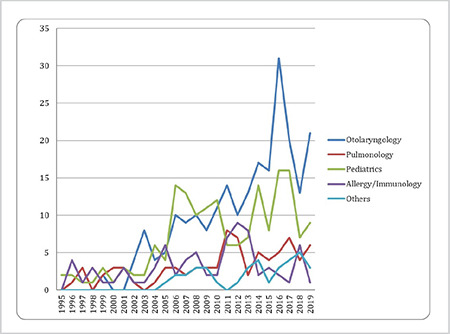
Distribution of “allergic rhinitis” publications from Turkey in PubMed^®^ according to the first author’s affiliation and years

**Figure 2:**
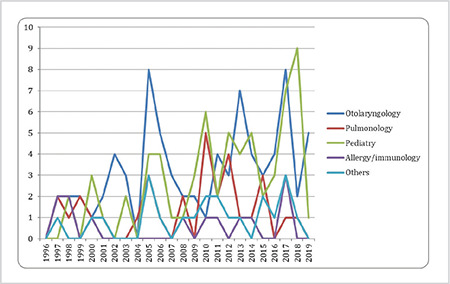
Distribution of “allergic rhinitis” publications from Turkey in TRDizin^®^ according to the first author’s affiliation and years

“Guidelines/consensus” articles were not included in the specialty group comparison given that these articles involve high numbers of authors from different specialties and their names are listed in alphabetical order (Figures 1, 2 and Table 1). Some of the publications in PubMed^®^ also appeared in TRDizin^®^, and these were not removed from either of the indices but counted under both.

For specialty group comparisons, all articles were grouped under “Otorhinolaryngology (ORL),”  “Pediatrics,” adult “Pulmonology” and adult “Allergy/immunology ” and listed as “Others” if the first author did not fall under any of these categories. The classification of each publication was determined based on the affiliation of the first author of the study, with the department of the first author garnered from the Affiliations tab in the PubMed^®^ database. When the specialty of the first author could not be ascertained using this tab, the affiliation was established by accessing the full text of the article or other publications by the author. In TRDizin^®^, the first author’s affiliation was determined by accessing the full text of the identified article.

A review of affiliations revealed the usage of various terms for pulmonology, pediatrics, and adult allergy/immunology. Of these, Chest Medicine, Pulmonary Diseases (Dis.), Chest Dis. Department (Dept.), Allergic Dis Sub-Dept., Pulmonary Dis.-Allergy Dept., Dept. of Chest Dis., Division (Div.) of Immunology and Allergy, Dept. of Pulmonary Dis.-Allergy, Dept. of Chest Dis. Adult Allergy Unit, and Div. of Allergy and Immunology Dept. of Chest Dis. were classified under the “Pulmonology” group; Dept. of Pediatrics, Dept. of Pediatric Allergy, Dept. of Paediatrics, Pediatric Allergy and Asthma Unit, Dept. of Pediatrics Div. of Allergy, Pediatrics Dept. of Allergy, Dept. of Pediatric Pulmonology, Paediatrics Div. of Allergy and Pulmonology, Dept. of Pediatric Allergy and Pulmonology, Div. of Pediatric Allergy and Pulmonology, Dept. of Pediatrics, Div. of Allergy/Immunology, Dept. Pediatric Allergy and Asthma, Dept. of Pediatrics, Div. of Allergy and Chest Dis. were classified under the “Pediatrics” group; and Internal Medicine, Allergy and Clinical Immunology Unit, Dept. of Clinical Immunology and Rheumatology, Div. of Allergy and Clinical Immunology, Div. of Allergy and Immunology, Internal Medicine Dept. of Allergy, Internal Medicine, Div. of Allergy, Dept. of Immunology, Allergy Div. Dept of Internal Medicine, Dept. of Allergic Dis., Dept. of Internal Medicine, Div. of Immunology and Allergy, Dept. of Internal Medicine and Immunology Div. of Immunology and Allergy, Allergy Div., Dept. of Internal Medicine were classified under the “Allergy/Immunol” group. Table 1 presents the distribution of publications by affiliations. The “Others” group, under which the publications with a first author that did not fall into any of the four groups, consisted of anesthesiology, biochemistry, biology, cardiology, child and adolescent psychiatry, dermatology, endocrinology, environmental engineering, family medicine, gastroenterology, ophthalmology, medical biology, microbiology, molecular biology, nuclear medicine, nursing, occupational medicine, parasitology, pediatrics and infectious dis., pharmaceutical microbiology, pharmaceutical technology, pharmacy, pathology, physiology, rheumatology and urology.

### Statistical Analysis

All statistical analyses were performed using the SPSS 25.0 software (IBM SPSS Statistics 25 [Armonk, NY, USA: IBM Corp.]). Categorical data were reported as number and percent. We used significance test for a difference in two proportions. In the interpretation of the comparison results, the Bonferroni correction was used to avoid a decrease in our confidence level. Alpha values ​​as much as the number of paired comparisons (10 paired examinations were made) were arranged (0.05/10=0.005) and significance was interpreted according to this alpha level.

Approval was received from the Ethics Committee of Pamukkale University (60116787-020/71420).

## Results

The number of publications identified was 624 in PubMed^®^ and 213 in TRDizin^®^. Table 1 shows the distribution of publications by their first author’s affiliation. Review of the number of publications in both the PubMed^®^ and the TRDizin^®^ databases identified the largest number of publications in the ORL group, followed by the Pediatrics group (Table 1). “Guidelines/consensus” articles were not included in the specialty group comparison given that these articles involve high numbers of authors from different specialties and their names are listed in alphabetical order. Table 2 shows the distribution of the number of publications by types of study and Table 3 shows the breakdown of the types of publications by five-year periods to provide further insight into the trend in the numbers over the 25-year period of our study.

Figures 1 and 2 show the distribution of publications over a 25-year period in the PubMed^®^ and TRDizin^®^ databases by the defined groups and years. The analysis done to identify whether there were any statistical differences among the groups in terms of the total number of publications identified in PubMed^®^ over the 25-year period, the number of articles was found statistically higher in the ORL group than in the Pulmonology (p=0.0001*, p<0.05) and Allergy/immunology (p=0.0001*, p<0.05) groups.

The analysis done to identify whether there were any statistical differences in the total number of publications identified in TRDizin^®^ among the groups over the 25-year period revealed that the number of articles was also statistically higher in the ORL group than in the Pulmonology (p=0.0001*, p<0.05) and Allergy/Immunology (p=0.0001*, p<0.05) groups, while there was no statistical difference between the ORL and Pediatrics groups in terms of the number of publications (p>0.05).

## Discussion

AR and otolaryngic allergy are conditions that are diagnosed and treated by otolaryngologists, and these treatment methods have undergone continuous development since the advent of the ORL specialization in Turkey (7). The 1980s witnessed an increasing interest in otolaryngic allergy and AR, and the epidermal skin test and immunotherapy practices that were developed in the 1980s gained momentum in the 2000s (7). In the 2000s, allergy outpatient clinics began to spring up, managed by otolaryngologists, where epidermal skin tests, allergy treatments and immunotherapies were commonly performed. In 2013, such practices were interrupted after hospital administrators started preventing otolaryngologists from providing epidermal skin test and immunotherapy services on the grounds that they were not covered by the Social Security Institution (SSI). Another problem was that AR could be considered within the fields of multiple specialties, diagnosed and treated by ORL, pulmonology, clinical immunology and pediatric departments, and working with the same types of patient groups could sometimes lead to disputes among specialties.

Academic otolaryngologists do research as part of their job, just as other academicians do in their areas of interest and present their research results at congresses and publish in scientific journals to share their findings with the scientific community. Have the SSI practices, which were brought into force in 2013 and partially improved upon in 2016, had any effect on scientific research and the number of publications made? The present study identified an increasing number of publications with otolaryngologists as their first authors since 1994, although no publications were made in certain years, and this was applicable to both the international PubMed^®^ and national TRDizin^®^ databases (Figures 1, 2). It was further ascertained that the restrictive regulations applied to otolaryngologists by the SSI related to AR had no effect on the research and publications on AR. That the academic interest of otolaryngologists in AR has undergone a steady growth over the years suggests that academic otolaryngologists who have taken an active interest in AR at an academic level despite the challenges faced in practice have continued to carry out scientific studies. That said, in Turkey, academicians publish articles not just to contribute to their area of interest, but also to secure academic promotions. It is probable that researchers, although they were not academically interested or had no plans to work in this field, may have selected this field for study because they frequently encounter AR patients.

Another finding of the present study was that the publications on AR were mostly based on patient data (Table 2); in other words, all relevant specialties were seen to have focused on epidemiological and clinical practice rather than experimental laboratory research. It can thus be said that the researchers have had ongoing contact with AR and AR patients over the 25-year period. An increase, however, was seen in the number of experimental studies over the last 10 years of the study period (Table 3), and this suggests that researchers with an interest in AR are now also engaging in experimental studies.

Another finding of the presented study was that there are a growing number of publications in the literature that were jointly written by many authors from different fields of specialty, such as guidelines or international expert consensus documents (guidelines/consensus) on AR, in the recent years (Table 3). These publications aim at devising international guidelines to steer the building of a common language to be used by all physicians around the world, or to declare consensus as was the case with “Allergic Rhinitis and its Impact on Asthma” (8). Such publications essentially contribute to the development of common guidelines and solutions from a universal perspective, with the cooperation of authors from the different countries around the world. Several authors from Turkey have taken part in these teams, which is clear evidence of the international recognition of our country in the field of AR. There has been a remarkable increase in the number of such mentioned publications in the last five years of the study period (Table 3); however, since these publications cannot be defined as review or research paper, they have been classified under a separate publication category as guidelines/consensus.

An examination of Table 3 reveals a remarkable increase in the numbers of reviews and experimental research papers from Turkey in the last five years, with all (100%) experimental studies and 90% of the reviews conducted over the last 10 years of the study. Based on this finding, it can be suggested that researchers with an interest in AR concluded their clinical research and started to work on experimental studies. Another reason for this finding may be the increasing opportunities that open up in experimental research courses and in experimental research laboratories (9). Regarding the increased number of reviews, it can be suggested that researchers interested in AR have gained sufficient experience and conducted reviews as a means of communicating their own experiences.

The most interesting finding of our study was the distribution of the total number of publications on AR in the 25-year period by the specialties involved in AR (Figures 1, 2 and Table 1). Accordingly, an examination of the total numbers over the 25 years revealed that not only did the number of publications on AR by academic otolaryngologists increase over the years (Figures 1, 2), but were also statistically higher than those authored by the other two specialties (Table 1). Based on this finding, we can suggest that otolaryngologists have a greater academic interest in AR and otolaryngic allergic diseases than the other two specialties involved in adult AR. The natural cause of this may be the fact that patients with AR tend to consult otolaryngologists when experiencing nasal complaints rather than other specialties.

One limitation of the presented study was its failure to include academicians from all related specialties. The number of medical faculties is increasing every year in Turkey, leading to an increase in the number of academicians, and this may also be behind the increasing number of publications on AR (10). Another limitation was that the study assessed only the number of publications, with no qualitative analysis of their content. An analysis of all AR publications by relevant specialties in our country identified no studies in “The top 100 most influential articles in AR from 1970 to 2018” (11). Based on the previous studies analyzing the quality-related problems of the publications from Turkey and looking into their scientific contributions, it can be suggested that these problems may be attributable to several reasons, such as the prevalence of publications made for only academic promotion purposes, the lack of research infrastructure, the low level of research funding/support and the lack of a research tradition (10). All of these topics, however, fall outside of the scope of the presented study and should be examined in further studies.

## Conclusion

The analysis of the number of 25-year AR publications from Turkey revealed that the academic interest of otolaryngologists in AR was unaffected, despite the challenges experienced in practice, with an increasing number of publications noted. When the number of 25-year publications was examined, ORL recorded the highest number of publications among all specialties.

**Main Points**

• Academic interest of otolaryngologists in research of allergic rhinitis was unaffected, despite the challenges experienced in practice.

• The number of allergic rhinitis publications by otolaryngologists has increased steadily over the 25-year period.

• Otorhinolaryngologists recorded the highest number of allergic rhinitis publications among all specialties in the 25 years.

## Figures and Tables

**Table 1:**
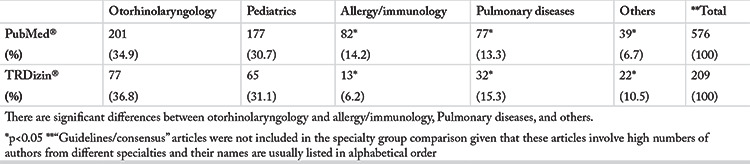
Distribution of the total numbers of publications listed in PubMed^®^ and TRDizin^®^ over the 25-year period according to the first author’s affiliation

**Table 2:**
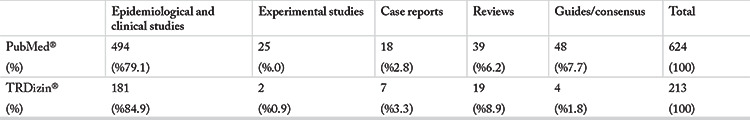
Distribution of the total number of publications in PubMed^®^ and TRDizin^®^ over the 25-year period according to the article type

**Table 3:**
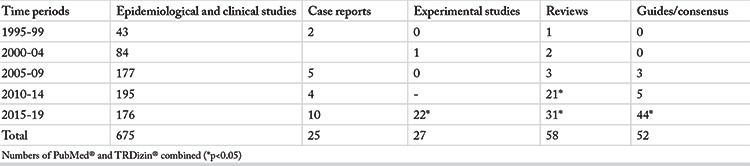
Distribution of publication types by five-year periods
